# Directed Evolution of *Pseudomonas fluorescens* Lipase Variants With Improved Thermostability Using Error-Prone PCR

**DOI:** 10.3389/fbioe.2020.01034

**Published:** 2020-09-02

**Authors:** Lijun Guan, Yang Gao, Jialei Li, Kunlun Wang, Zhihong Zhang, Song Yan, Nina Ji, Ye Zhou, Shuwen Lu

**Affiliations:** Institute of Food Processing, Heilongjiang Academy of Agricultural Sciences, Harbin, China

**Keywords:** lipase, structural analysis, site-directed mutagenesis, thermostability, methanol tolerance

## Abstract

Lipases catalyze the hydrolysis of fats and oils, and have been widely used in various industrial fields. However, bacterial lipases have a lower thermostability in industrial processes, which was a limiting factor in their industrial application. In this study, we obtained an improve variant of *Pseudomonas fluorescens* lipase (PFL) with enhanced thermostability using classical error-prone PCR. Wild-type PFL showed an optimal temperature and pH of 50°C and pH 7.5, respectively. Due to the low thermostability of PFL, a library containing over 3000 individual mutants as constructed using error-prone PCR. Screening for thermotolerance yielded the mutants L218P and P184C/M243C with *T*_m_ values of 62.5 and 66.0°C, which was 2.5 and 6°C higher than that of the WT, respectively. The combination of the two mutants (P184C/M243C/L218P) resulted in an approximately additive effect with a *T*_m_ value of 68.0°C. Although the increase of *T*_m_ was not substantial, the mutant also had dramatically increased methanol tolerance. Structural analysis revealed that the introduction of a disulfide bond between P184C and M243C and the substitution of Pro to reduce the flexibility of a loop increased the thermostability of PFL, which provides a theoretical foundation for improving the thermostability and methanol tolerance of lipase family I.1 to resist the harsh conditions of industrial processes.

## Introduction

Lipases (triacylglycerol-ester-hydrolases, EC 3.1.1.3) are ubiquitous enzymes that catalyze the synthesis of esters and the release of fatty acids via the hydrolysis of fats and oils. Lipases have been widely used in various industrial fields, such as the food industry, paper manufacturing, detergent industry, pharmaceuticals, cosmetics, bioremediation, textiles, waste oil disposal, and biodiesel production (Cai et al., 2011; Fickers et al., 2011). Lipases are ubiquitously produced by microorganisms, animals, and plants. However, most lipases widely used in industry are derived from microbial sources, because the respective genes are relatively easy to access, can be efficiently expressed, and inexpensively produced by large-scale fermentation (Sharma et al., 2002). Recently, many lipases from microbial sources, such as *Bacillus subtilis*, *Yarrowia lipolytica*, *Penicillium expansum*, *Pseudomonas* species, *Bacillus licheniformis*, *Aspergillus niger*, and *Candida rugosa*, have been characterized. However, many lipases from these microorganisms showed low thermostability and unsatisfactory stability in industrial processes, which was a limiting factor for their industrial application. In addition, high temperatures would be helpful to increase reaction rates and substrate solubility and to reduce contamination by environmental microorganisms (Heater et al., 2019). Thermostability of the enzyme is also a prerequisite for industrial processes that rely on high enzyme activity, mass transfer, reactant solubility, fine chemical synthesis and so on (Sharma et al., 2002). Lipase with higher thermostability play a vital role in industrial applications, and it is urgent to improve the thermostability of unstable lipases (Wen et al., 2012; Miyakawa et al., 2015).

Many strategies to date have been developed to improve the thermostability of enzymes with varying degrees of success. Among these approaches, random mutagenesis is most practical when coupled with an efficient screening or selection procedure to identify clones expressing variant enzymes with properties of interest, such as increased activity, substrate specificity, stability, and stereoselectivity (Reetz, 2004; Eom et al., 2005; Heater et al., 2019; Huang et al., 2019). Chen et al. found that the lipase triple mutant D60N/Q103R/N218S, which was obtained through random mutagenesis, was more active in the presence of dimethylformamide (DMF) (Chen and Arnold, 1991). Huang et al. (2019) improved the alkali stability of *P. cyclopium* lipase, and the mutant N157F obtained using error-prone PCR was 23% more stable at pH 11.0 than the WT. The half-life of the purified mutated Lip2 lipase from *Y. lipolytica* at 60°C was 127-fold increase compared to the WT enzyme (from 1.5 min to 3 h) by error-prone PCR (Bordes et al., 2011). Thermostable lipases are highly sought after due to their wide uses in many industrial applications. Therefore, protein engineering including directed evolution and rational design according to the molecular structures should be applied to improve the thermostability of lipases.

Here, we describe the expression, characterization, and directed revolution of a lipase from *P. fluorescens*, named PFL. Mutant PFL with enhanced thermostability were selected and the purified enzymes were further characterized. The mutants of L218P and P184C/M243C showed *T*_m_ values of 62.5 and 66.0°C, which was 2.5 and 6°C higher than that of the WT, respectively. Structure analysis revealed the thermostability mechanism of PFL at the molecular level and provides a theoretical basis for the development of highly thermostable lipases. Notably, the mutant also has a dramatically increased methanol tolerance. This study provides valuable information on lipases form family I.1, which is the least known family of bacterial lipases.

## Materials and Methods

### Chemicals and Enzymes

The artificial substrates with different carbon-chain length (C2-C16), including *p*-nitrophenyl butyrate (C4), *p*-nitrophenyl caproate (C6), *p*-nitrophenyl caprylate (C8), *p*-nitrophenyl caprate (C10), *p*-nitrophenyl laurate (C12), *p*-nitrophenyl myristate (C14), and *p*-nitrophenyl palmitate (C16), were obtained from Sigma-Aldrich (St. Louis, MO, United States). Restriction enzymes were purchased from TaKaRa (Dalian, China). The KOD-Plus-Mutagenesis Kit and KOD-Plus-Neo Kit were purchased from Toyobo (Japan). Other reagents and chemicals used in this study were sourced from local vendors and were of analytical grade.

### Cloning and Expression of the PFL Gene

The sequence of PFL (Protein ID: AAC15585.1) was codon-optimized for *E. coli* and synthesized by Genewiz (Suzhou, China). The resulting product was inserted into the vector pET22b (+) (Novagen, Madison, WI, United States) using the *Nde*I and *Eco*RI sites with T4 DNA Ligase (NEB, Ipswich, MA, United States) following the protocol, resulting in the pET-PFL plasmid containing the PFL gene. Then, pET-PFL was introduced into *E. coli* BL21(DE3) by heat shock (42°C for 45 s), which was cultivated in Luria-Bertani (LB) containing 10 g/L tryptone, 5 g/L yeast extract, and 5 g/L NaCl with 100 μg/mL of ampicillin at 37°C. Isopropyl β-D-1-thiogalactopyranoside (IPTG) was added to a final concentration of 0.5 mM when the optical density at 600 nm (OD_600_) reached 0.6–0.8 after cultured at 37°C for about 2 h, followed by incubation at 16°C for 20 h to express the PFL.

### Purification of PFL Enzyme

The cells carrying PFL were harvested by centrifugation at 5,000 × *g* and resuspended in lysis buffer [20 mM Tris-HCl pH 8.0, 20 mM imidazole, 500 mM NaCl, and 1 mM dithiothreitol (DTT)], after which 1 mM phenylmethanesulfonylfluoride (PMSF), and 1 mg/mL lysozyme were added to the solution. The cells were disrupted by sonication (SCIENTZ-950E, Ningbo Scientz Biotechnology Co. Ltd., Ningbo, China) on ice. The resulting crude lysate was cleared by centrifugation at 40,000 × *g* and 4°C for 30 min. The His-tagged enzyme in the cleared supernatants was trapped on Ni-NTA Superflow resin (Qiagen, Hilden, Germany) and loaded into an open column. The resin was washed twice with 10 column volumes wash buffer (20 mM Tris-HCl pH 8.0, 30 mM imidazole, 500 mM NaCl, and 1 mM DTT) and then eluted with 10 mL elution buffer (20 mM Tris-HCl pH 8.0, 300 mM imidazole, 0.5 M NaCl, and 1 mM DTT). The eluates were dialyzed against 20 mM MES pH 6.5 containing 1 mM DTT overnight and further purified by loading on a Source 15S ion-exchange column (GE Healthcare, United States). The column was eluted with a gradient of 0–1 M NaCl in 20 mM MES (pH 6.5) with 1 mM DTT, followed by size exclusion chromatography on a Superdex 200 10/300 GL (GE Healthcare) in 20 mM Tris-HCl (pH 8.0) with 0.15 M NaCl and 1 mM DTT. The resulting eluate containing PFL was used for further activity assays. The protein concentration was measured using a BCA assay kit (Solarbio, Beijing, China) following the manufacturer’s protocol, and the purity of the lipase was assessed by SDS-PAGE with Coomassie Brilliant Blue R-250 staining followed by densitometric analysis using Image Lab Software (Bio-Rad, Hercules, California, United States).

### Enzyme Activity Assay

The enzyme activity was measured spectrophotometrically on a microplate reader (SpectraMax i3x, Molecular Devices, CA, United States) using *p*-nitrophenyl caprylate as substrate. One unit of enzyme activity was defined as the amount of enzyme that releases 1 μM *p*-nitro-phenol per min under the optimal reaction conditions (50°C, pH 7.5). All experiments were carried out in triplicates, and the data are shown as the means ± SD.

The optimal reaction temperature for the purified lipases were determined between 20 and 70°C, and the optimal pH in the range of 5.5–10. The pH stability of the purified enzyme was determined by incubating it at 4°C for up to 2 h. The thermostability of the lipases was calculated by incubating it in 20 mM phosphate buffer (pH 7.5) at different temperatures (20–70°C) for 30 min, and the relative residual activity of each enzyme solution was calculated using the same assay conditions as above. The effects of various metal ions and organic solvents on the activity of the lipases were investigated using the purified enzyme solution.

The activity assay was the same except for the addition of 5 mM EDTA, Ca^2+^, Mg^2+^, Co^2+^, Ni^2+^, Fe^2+^, Zn^2+^, Cu^2+^, or Mn^2+^. The wild-type and mutated lipases were incubated for 120 min at 4°C in aqueous medium with different solvents: methanol, ethanol, dimethylformamide (DMF), dimethyl sulfoxide (DMSO) and acetonitrile (10%, v/v), and the relative residual activity for each enzyme was calculated using the same assay conditions as above. The maximal lipase activity at 50°C was defined as 100%.

Substrate specificities of the purified lipases were determined in assay mixtures containing substrate with various carbon-chain lengths: *p*-nitrophenyl butyrate (C4), *p*-nitrophenyl caproate (C6), *p*-nitrophenyl caprylate (C8), *p*-nitrophenyl caprate (C10), *p*-nitrophenyl laurate (C12), *p*-nitrophenyl myristate (C14), and *p*-nitrophenyl palmitate (C16).

The half-life time of the WT and mutant at different temperature were determined using the first-order inactivation kinetic model at pH 7.5, while the half-life time of the WT and mutant at different pH were determined at 50°C.

All assays were done in triplicate, and the data are shown as the means ± SD.

### Circular Dichroism (CD) Spectroscopy

To assess the secondary structure, far-UV CD spectra in the range of 190–260 nm were recorded using MOS-450 CD spectropolarimeter (Biologic, Claix, Charente, France) with a 1 mm path-length cell at 25°C. Four scans were recorded using a bandwidth of 0.1 nm, a step resolution of 0.1 nm, and a scan rate of 1 nm/s, and averaged for each spectrum. The protein was dissolved in 20 mM phosphate buffer pH 7.5 at a final concentration of 0.02 mg/mL. Analysis of the protein secondary structure was performed using SELCON3 software^1^ (Whitmore and Wallace, 2008).

### Differential Scanning Fluorimetry

Differential scanning fluorimetry (DSF) experiments were performed using a CFX96 Real-Time PCR system (Bio-Rad, Hercules, CA, United States) at an excitation wavelength of 492 nm and emission wavelength of 610 nm (Niesen et al., 2007). The samples were prepared in microplate wells containing 22.5 μL of 5 μM purified PFL protein, 1 mM DTT, and 2.5 μL of a 25-fold dilution of SYPRO Orange (reporter dye; Invitrogen, United States). Then, the samples were sealed with Optical-Quality Sealing Tape and heated in a CFX 96 Real Time PCR System using a linear 30–95°C gradient at a rate of 0.5°C per 10 s. The inflection point of the fluorescence vs. the temperature curves was identified by plotting the first derivative against temperature in CFX Manager Software (Bio-Rad, Hercules, CA, United States). The *T*_m_ value was defined as the minimal peak of the fluorescence curve.

### Construction of the PFL Mutant Library Using Random Mutagenesis

The PFL mutant library was constructed based on pET-PFL as the template using the GeneMorph II Random Mutagenesis Kit (Agilent Technologies, Texas, TX, United States), according to the manufacturer’s protocol. The PFL gene sequence was amplified using the primers PFL_F (GGAATTCCATATGTCACAGTCAACAGCTACACGC) and PFL_R (GGAATTCAAGGCCAGCAGCTTTAAGGCG). The products of error-prone PCR were inserted into the vector digested with *Nde*I and *Eco*RI, resulting in the pET-MPFL plasmid library. The library was then introduced into *E. coli* BL21(DE3) cells for protein expression.

The individual mutants, more than 3000 clones, comprising the error-prone PCR library, were spread on LB agar plates with appropriate antibiotic and incubated overnight. The cells were then moved to LB agar plates containing 0.3% tributyrin and 0.5 mM IPTG. The colonies surrounded with various degrees of clearing were selected and cultured in 96-well plates with 200 μL of LB medium containing 100 μg/mL of ampicillin at 37°C overnight. Then, 5 μL of the mutant cultures were transferred into new 96 deep-well plates with fresh LB medium containing 100 μg/mL of ampicillin, and incubated until the OD_600_ reached 0.6–0.8, followed by the addition of IPTG at a final concentration of 0.5 mM to introduce the protein overexpression at 16°C for 18 h. The cells were harvested by centrifugation at 4,500 × g for 15 min and resuspended in enzyme activity assay buffer containing appropriate lysozyme and BugBuster (Novagen, MA, United States). The supernatant was collected by centrifugation as above after incubation at 60°C for 30 min with shaking, and the clear supernatants were used to determine the residual activity of mutant enzymes at 50°C using the *E. coli* BL21(DE3) harboring the vector pET22b (+) as control. The positive mutants with improved thermostability were selected for further analysis.

### Site-Directed Mutagenesis

Site-directed mutagenesis was conducted using the KOD-Plus-Mutagenesis Kit (Toyobo, Japan) on plasmid pET-PFL containing the PFL gene as the template using the primers F: CCTTTCGACGGTACAAACCGCTCATGTCGCC, and R: GTTGCCGCCGCGGTCGGTCTTGCCAG. After analyzing the PCR reaction by agarose gel electrophoresis, the template DNA was digested using *Dpn*I for 60 min at 37°C, and the products were ligated using T4 Polynucleotide and Ligation kit (NEB, Ipswich, MA, United States) according to the manufacturer’s protocol. *E. coli* JM109 competent cells were transformed with the ligation product for DNA cloning, and the constructs were verified by sequencing (BGI, China). The obtained plasmids were introduced into *E. coli* BL21 (DE3) competent cells for protein expression as above.

### Structural Modeling of PFL

The homology model of PFL was generated using Swiss-Model^2^ (Biasini et al., 2014) with the crystal structure of *Pseudomonas aeruginosa* lipase (PDB ID: 1EX9) as the template (Nardini et al., 2000; Aschauer et al., 2018). VERIFY-3D was used to determine the compatibility of the atomic model (3D) with the amino acid sequence (1D)^3^ (Lüthy et al., 1992). The generated model structure of PFL was rendered and analyzed using PyMol^4^ (Humphrey et al., 1996).

## Results and Discussion

### Bioinformatic Analysis

**FIGURE 1:**
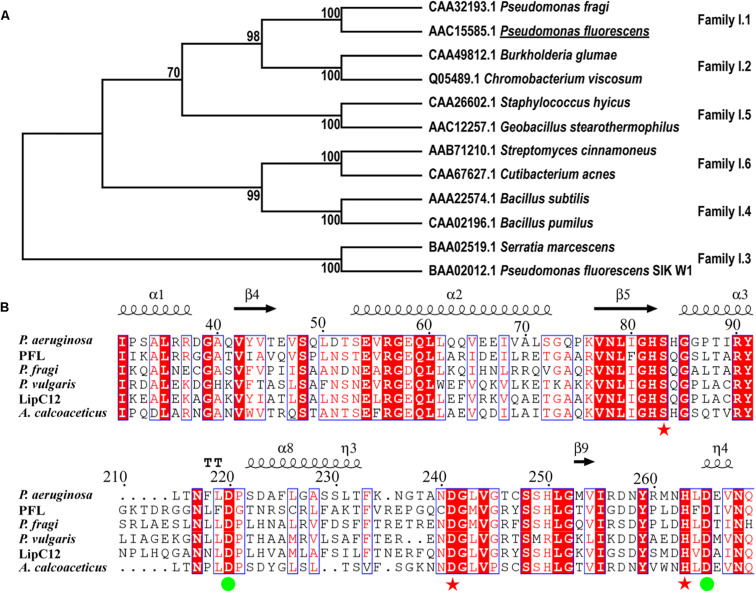
Phylogenetic tree of PFL and other enzymes from lipase family I **(A)**, multiple amino acid sequence alignment of the lipase family I.1 **(B)**, the residues constituting the catalytic triad are marked with red asterisks, residues constituting the Ca^2+^-binding site are marked with green circles.

A search for lipases from family I of true lipases was performed using BLAST. The identified homologous proteins were compared with PFL based on their amino acid sequences (Figure 1A). The phylogenetic analysis showed that PFL belongs to family I.1, and shares higher sequence identities with family I.2, which had a slightly smaller size (30–32 kDa) than family I.1 (33 kDa) due to the deletion of several amino acids forming an anti-parallel β-strand on the surface of the lipase. By contrast, the lipases family I.3, family I.4, family I.5 and family I.6 had molecular masses in the range of 50–60, 20, 45, and 75 kDa, respectively. The lipases from family I.3 did not contain the N-terminal signal peptide and Cys residues (Arpigny and Jaeger, 1999). The lipases from family I.1 shared over 40% sequence identity with family I.2, but their properties such as enantioselectivity, thermostability, and solvent tolerance were different. There are two Asp residues involved in the Ca^2+^-binding site of lipases from subfamilies I.1 and I.2 of true lipases, which are believed to be vital in the stabilization of these enzymes (Figure 1B). The residues of the catalytic triad (S83, D241, and H263) were also conserved, as expected.

### Characterization of Purified Lipase

**FIGURE 2:**
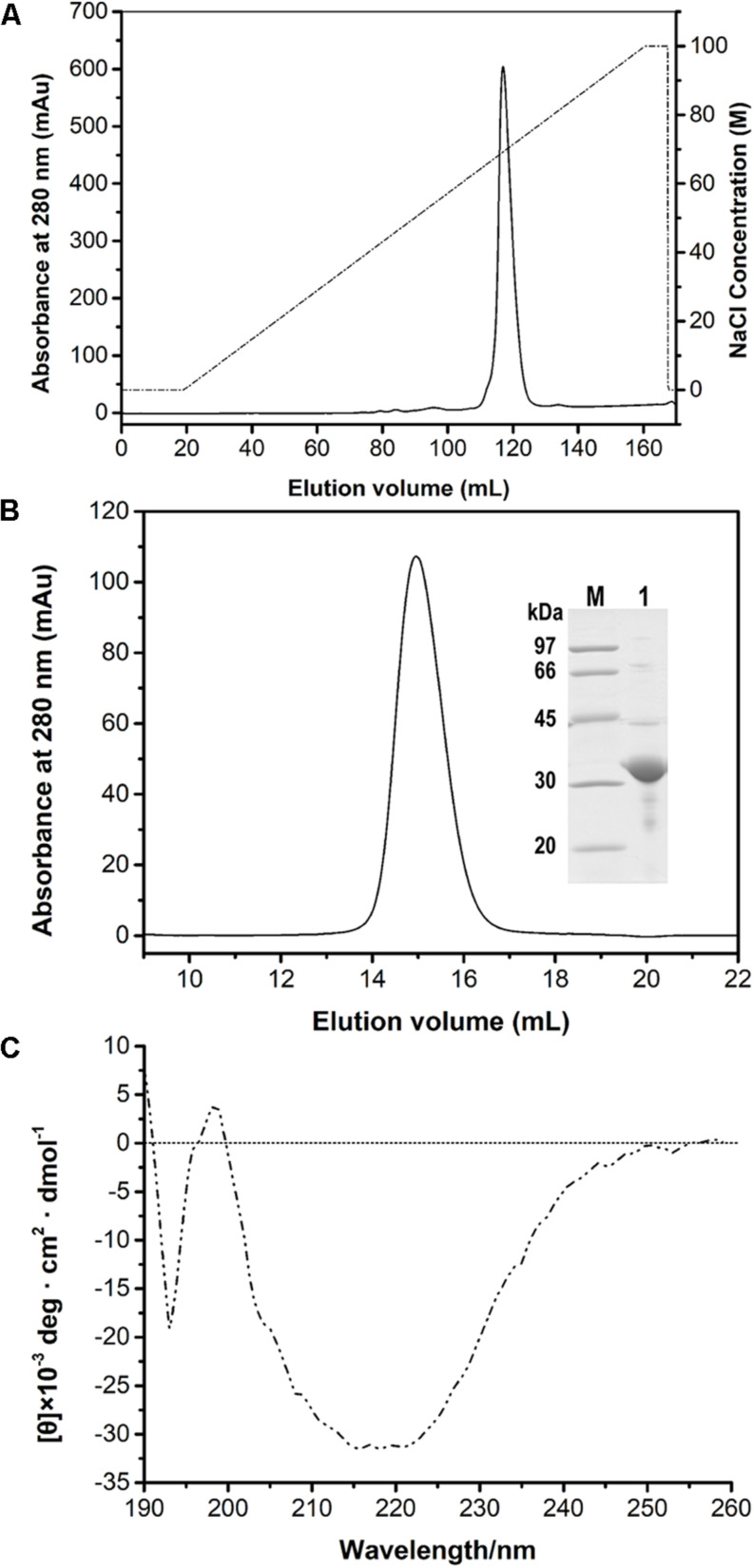
Purification of PFL by anion-exchange **(A)** and size-exclusion chromatography and SDS-PAGE analysis **(B)**. Lane M, protein molecular mass markers; lane 1, purified PFL after size-exclusion chromatography visualized by Coomassie blue staining. CD spectrum of PFL **(C)**.

After the induction of recombinant *E. coli* BL21 (DE3) cells, recombinant PFL was detected in the cell-free supernatant, which indicated that PFL was successfully overexpressed. For purification, the supernatants after cell lysis were purified using a Ni-NTA Superflow column, followed by anion-exchange chromatography (Figure 2A). A single major peak at 280 nm was observed using size exclusion chromatography (Figure 2B). The yields and purities of PFL after different stages of the purification are summarized in Table 1. Finally, 4.39 mg of PFL with high purity (96.7%) was efficiently obtained from 100 mL of cell culture.

**TABLE 1:** Summary of lipase at different stages of the purification process.

**Purification step**	**Total protein (mg)**	**Target protein (mg)**	**Purity (%)**	**Yield (%)**
Crude	86.25	20.53	23.8	100.0
Eluate from Ni-NTA resin	15.32	12.30	80.4	59.9
Eluate from Source S column	9.81	8.73	89.0	42.5
Eluate from Superdex 200 10/300 GL column	4.54	4.39	96.7	21.4

*The results are based on cell material from a 100 mL *E. coli* culture.*

Far-UV CD spectra in the range of 190–260 nm were recorded to determine the secondary structure of PFL. Figure 2C shows a strong positive absorption peak at 196–197 nm, which is a typical feature for α-helices, whereas a negative absorption peaks centered around 192–194 and 215–220 nm are a typical feature β-sheet structures (Liu et al., 2017). The secondary structure percentages of PFL obtained from CD spectra were 18.6% α-helix, 37.9% β-sheet, 9.2% β-turn structures, and 34.6% unstructured regions.

**FIGURE 3:**
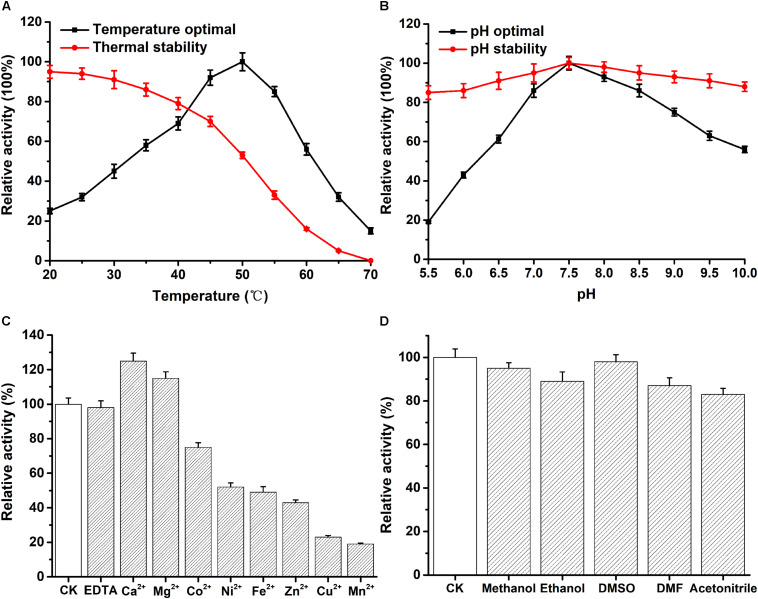
Effect of temperature and pH on the enzyme activity of PFL. **(A)** temperature dependence and thermostability. **(B)** pH dependence and stability. The effect of mental ions **(C)** and organic solvents **(D)** on the enzyme activity of PFL. All assays were repeated three times, and the data are shown as means ± SD.

The optimum temperature of recombinant PFL was 50°C (Figure 3A). As shown in Figure 3A, the enzyme stayed stable at low temperatures, and 80% of the enzyme activity was retained when incubated from 20 to 40°C for 30 min. However, the enzyme activity decreased to about 50% after PFL was incubated for 30 min at 50°C. Moreover, the effect of pH on PFL was studied, as shown in Figure 3B, PFL showed maximal activity at pH 7.5, and was determined to be stable at pH values between 7.0 and 8.5. The enzyme showed good stability for 2 h at different pH values, whereby the residual enzyme activity remained above 80% of the maximum activity, indicating that PFL has a very wide pH range, which are useful for potential industrial applications. Metal ions are important for thermostable enzymes (Sharma et al., 2002; Gupta et al., 2004). Therefore, we determined the effects of metal ions on PFL, 5 mM final concentrations of various metal ions were added and the activity of recombinant PFL was measured. As shown in Figure 3C, PFL was active without the addition of metal ions and in the presence of EDTA, indicating that it is not a metalloenzyme. However, the addition of Ni^2+^, Fe^2+^, and Zn^2+^ inhibited the enzyme activity strongly to around 50%, while Cu^2+^ and Mn^2+^ inhibited the enzyme activity by about 20%. By contrast, the presence of Ca^2+^ and Mg^2+^ had a positive effect, increasing the enzyme activity of PFL by 25 and 15%, respectively. These results were consistent with previous studies on other lipases, which found that Ca^2+^ often stimulates enzyme activity due to the formation of calcium salts of long-chain fatty acids (Gupta et al., 2004). Overall, the results suggest that PFL is not a metalloenzyme, which was also the case with other lipases (Sharma et al., 2002; Cai et al., 2011). PFL showed high resistance to various organic solvents, as shown in Figure 3D. PFL retained about 90% of initial activity in 10% (v/v) of different organic solvents, which indicated that PFL had good solvent stability. This is an important factor in synthesis reactions such as transesterification and esterification (Sharma et al., 2017).

**FIGURE 4:**
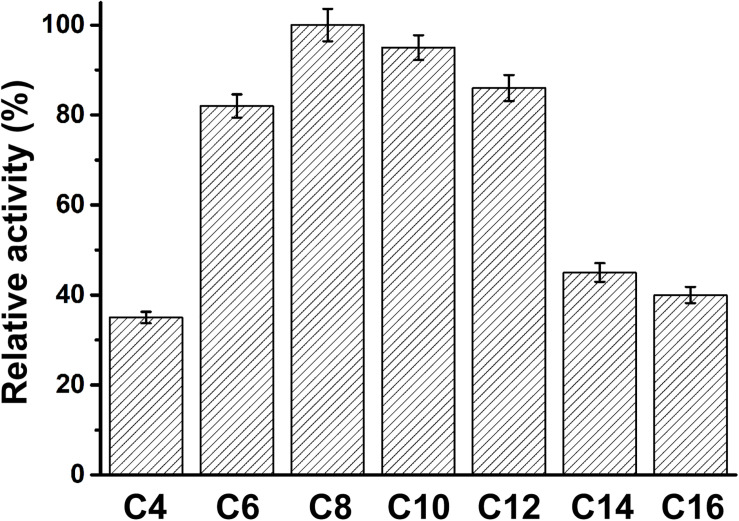
The substrate specificity of PFL, the relative activity of PFL toward *p*-nitrophenyl caprylate (C8) was set 100%. All assays were repeated three times, and the data are shown as means ± SD.

The specificity of PFL for substrates with various acyl chain lengths was also determined. The activity of PFL increased with increasing acyl chain length from C4 to C10, but decreased from C12, showing maximal activity with C8. However, the substrate specificity pattern of variants was broad, whereby *p*-nitrophenyl caprate was the optimum substrate, as shown in Figure 4.

### Screening the PFL Mutant Library

**FIGURE 5:**
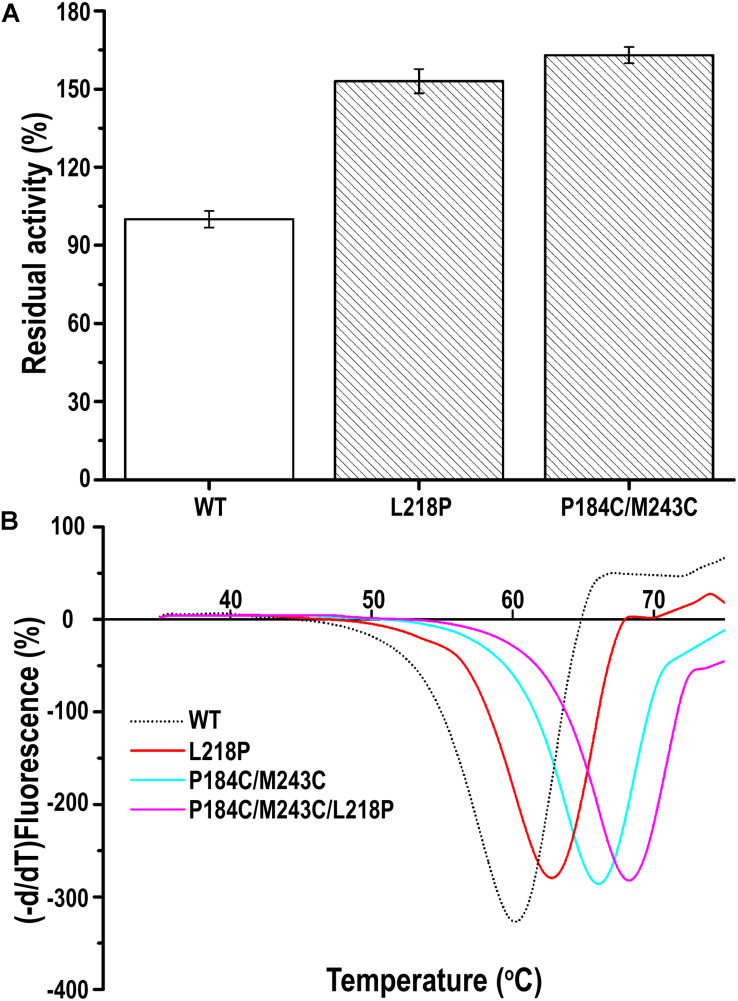
**(A)** The relative catalytic activity of mutant PFL variants, the substrate of *p*-nitrophenyl caprylate (C8) was used to assess activity of the mutants under optimal conditions. **(B)** Melting temperature curves of mutant and wild-type PFL in DSF experiments. The minimal values were considered to indicate the melting temperature of enzyme. All assays were repeated three times, and the data are shown as means ± SD.

A random PFL library containing over 3000 individual mutants was constructed through mutagenesis by error-prone PCR. The transformants were firstly selected on LB agar plates containing 0.3% tributyrin and 0.5 mM IPTG. The cells surrounded with various degrees of clearing, which indicates that the mutants showed activity after mutagenesis by error-prone PCR, were selected to decrease the workload. Among these transformants, two positive clones showed higher activity toward the substrate of p-nitrophenyl caprylate (C8) under optimal conditions following incubation at 60°C for 30 min than the wild-type PFL. As shown in Figure 5A, the residual activity of the two best mutants was respectively, 1.5- and 1.63-fold higher than that of the wild type after incubation at 60°C for 30 min. Then, the plasmids encoding the two mutants were isolated and verified. Sequencing revealed that the mutants had been mutated at one (L218P) or two (P184C/M243C) amino acid positions. Therefore, the mutants L218P and P184C/M243C were selected for further study.

### The Thermostability of PFL and Its Mutants

The thermostability of PFL and its mutants was characterized using differential scanning fluorimetry (DSF) experiments, which are widely used to determine the stability of enzyme due to its convenience (Niesen et al., 2007; Johnson et al., 2014). DSF experiments were also used to determine the thermostability of lipases in other reports. For example, Kim et al. determined that the melting temperature (*T*_m_) of the R249L mutant of *C. antarctica* lipase B (CalB) was 56.8°C, which was higher than that of the wild type. The mutant was developed using rational design based on B-factor calculations and Rosetta Design (Kim et al., 2010). Rodrigues et al. used DSF to determine the effect of 61 different ionic liquids on the stability of *Tl*L lipase from *Thermomyces lanuginosus* (Rodrigues et al., 2014). In this study, we used DSF to determine the stability of PFL with purified enzyme. The *T*_m_ values of the mutants L218P and P184C/M243C was increased from 60°C to 62.5 and 66.0°C, which was 2.5 and 6°C higher than that of the WT, respectively (Figure 5B). A combination of mutants with higher thermostability sometimes results in a cumulative effect (Liu et al., 2017). Therefore, the combined triple mutant P184C/M243C/L218P was generated using site-directed mutagenesis using the plasmid P184C/M243C as the template. As hoped, the triple mutant showed higher thermostability than either L218P or P184C/M243C, with an 8°C increase in the *T*_m_ value (68°C) compared to 60°C for wild-type PFL. Half-time of wild-type and mutant PFL was investigated at different temperature and pH, as shown in Table 2, the t_1/2_ of the mutants L218P, P184C/M243C, and P184C/M243C/L218P was increased to 1. 3-, 1. 9-, and 2.2-fold than that of wild-type, respectively. The t_1/2_ of the mutants at different pH also increased compared with wild-type of PFL.

**TABLE 2:** Half-life time (t_1/2_) of wild-type and mutant PFL at different temperature and pH.

**Enzyme**	**t_1/2_ (min)**				
**Temperature (°C)**	**20**	**30**	**40**	**50**	**60**
WT	139.63	89.81	67.46	32.56	18.32
P184C/M243C	272.60	174.20	129.26	64.25	38.36
L218P	183.47	128.81	92.15	43.51	25.91
P184C/M243C/L218P	392.54	232.15	149.25	73.12	48.62

**pH**	**6**	**7**	**8**	**9**	**10**

WT	18.59	27.35	29.42	22.75	15.43
P184C/M243C	44.42	52.40	61.27	42.10	26.85
L218P	24.16	38.25	42.60	30.67	19.20
P184C/M243C/L218P	46.91	67.48	69.51	59.40	46.75

### Structural Analysis of PFL and Its Mutants

**FIGURE 6:**
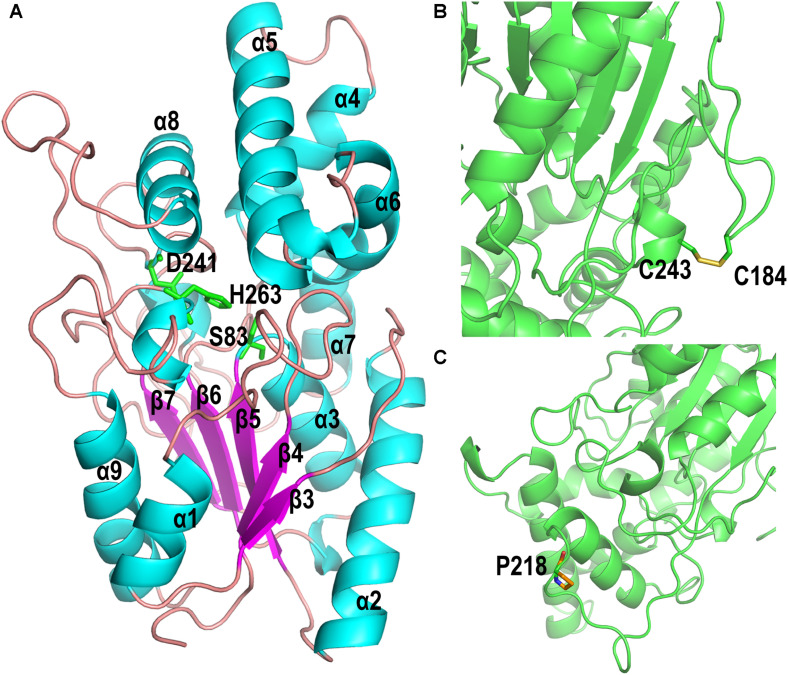
**(A)** Overall homology-based modeled structure of PFL. The residues constituting the catalytic triad are shown in green. **(B)** The introduction of the disulfide bond between P184C and M243C. **(C)** The substitution of Pro for Leu at position 218.

The homology model of PFL, which shows 47.2% identify with the lipase from *Pseudomonas aeruginosa* (PAL) (Nardini et al., 2000), was built online using Swiss-Model software. The homology model of PFL was analyzed using a Ramachandran plot, which indicated the favorable and allowed regions, respectively, accounted for 92.41 and 1.3% of the total, which suggested that the model had acceptable quality. As shown in Figure 6A, PFL has a typical α/β hydrolase fold with five β-strands in a parallel β-sheet surrounded by αβ-helices, and a lid domain with four α-helices as observed in many other homologous structures (Chopra et al., 2018; Martini et al., 2019). The conserved residues Ser83, Asp241, and His263 from the canonical catalytic triad of lipases. The structure of the lipases from family I.1 and family I.2 was similar, but there were major differences in the region following strand β7, where FPL did not contain the antiparallel β-sheet (b3 and b4) (Nardini et al., 2000).

The mutant P184C/M243C, which two has two additional cysteine residues, contained an intramolecular disulfide bond as shown in Figure 6B, which confirms earlier reports that the introduction of disulfide bonds can increase the thermal stability of lipases. The lipases from family I.1, which includes PFL, sometimes do not contain a disulfide bond, while all members of family I.2 contain a disulfide bond (Nardini et al., 2000). The introduction of disulfide bonds in lipases led to a considerable improvement of enzyme stability in many studies. For example, Han et al. (2009) increased the thermostability of *Rhizomucor miehei* lipase by introducing a novel disulfide bond. The thermostability of *Rhizopus chinensis* lipase was significantly improved by introducing a disulfide bond between F95C and F214C, with an 11-fold increase of the half-life at 60°C compared to the wild-type (Yu et al., 2012). Similarly, when a disulfide bond was introduced between A162C and K308C, the mutant *C. antarctica* lipase B (CalB) showed a 4.5-fold higher half-life at 50°C than that of the wild type (Le et al., 2012). Therefore, the introduction of a disulfide bond is an effective strategy for improving the thermal stability of lipases.

The variant lipase L218P increased the thermostability due to the L to P substitution on a long random coil (Figure 6C). Generally, proline residues in a polypeptide chain have less conformational freedom than other amino acids due to the restricted rotation of N-Cα in the pyrrolidine ring, which can decrease the entropy during protein unfolding (Zhou et al., 2010; Anbar et al., 2012). Therefore, the variant lipase L218P decreased the flexibility of loops and enhanced the rigidity of the long random coil at high temperature. Finally, the triple mutant P184C/M243C/L218P was constructed using site-directed mutagenesis to investigate the cumulative effect of the identified random mutations on the thermostability of PFL. The introduction of the disulfide bond between P184C and M243C increased the stability of the PFL molecule, while the substitution of proline reduced the flexibility of a loop and random coil without loss of enzyme activity. The introduction of a proline in a flexible region of an enzyme can improve its thermostability.

### Methanol Tolerance of PFL and Its Mutants

**FIGURE 7:**
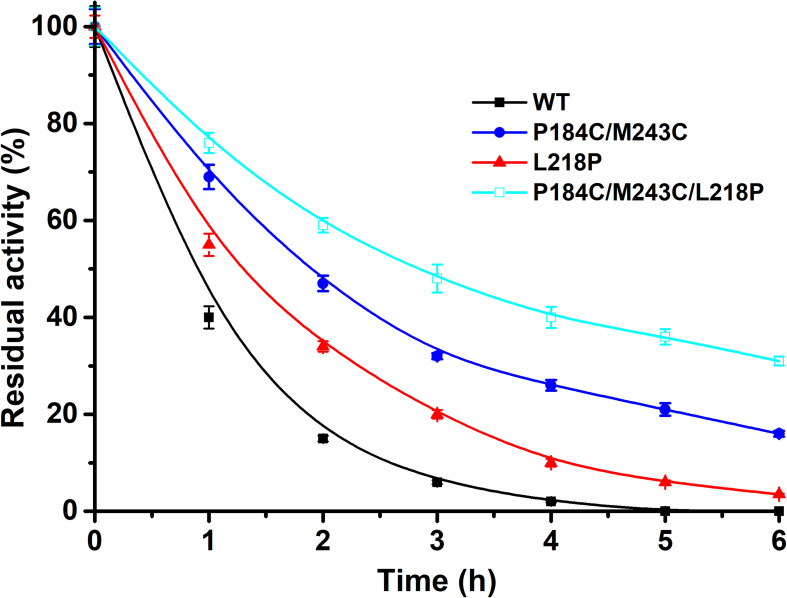
The residual activity of PFL and mutants in the presence of methanol after different incubation times. All assays were repeated three times, and the data are shown as means ± *SD*.

Methanol is widely applied for biodiesel production due to its low cost. Therefore, the tolerance to methanol of the mutants P184C/M243C, L218P, and P184C/M243C/L218P with purified enzyme was also determined. The residual activity was measured after 6 h of incubation in 50% methanol. As shown in Figure 7, the methanol tolerance of the mutants showed a significantly increase compared to the wild type. While the wild-type enzyme was inactive after 4 h of incubation, P184C/M243C, L218P, and P184C/M243C/L218P retained 32.0, 20.3, and 47.5% of their respective initial activities, which indicated that the mutants showed increased methanol tolerance. A previous study found a positive correlation between the solvent tolerance and thermostability of enzymes (Rasekh et al., 2014). This study therefore provides potentially useful lipase mutants with increased thermal stability and solvent tolerance.

## Conclusion

Variants of the PFL lipase from *P. fluorescens* with enhanced thermostability were obtained using the strategy of error-prone PCR. PFL showed optimal activity at 50°C and pH 7.5. It was stable and retained over 80% activity in the range of 20–40°C for 30 min. The activity of PFL was enhanced by Ca^2+^ and Mg^2+^, and it retained over 90% in 10% (v/v) of organic solvents. PFL showed broad substrate specificity, with maximum activity toward *p*-nitrophenyl caprylate (C8). A library containing over 3000 individual mutants was constructed through mutagenesis by error-prone PCR, and screening identified L218P and P184C/M243C with *T*_m_ values of 62.5 and 66.0°C, which was 2.5 and 6°C higher than that of the WT, respectively. The combination of the two mutants had a cumulative effect, with a *T*_m_ value of 68.0°C. The mutant also had dramatically increased methanol tolerance while retaining similar activity with that of the WT. Structural analysis of PFL revealed that the introduction of the disulfide bond between P184C and M243C and the substitution of proline increased the thermostability of PFL by reducing the flexibility of a loop and random coil, which provides a theoretical foundation and preliminary information on improving the thermostability of lipases from family I.1, enabling them to resist the harsh conditions of industrial processes.

## Data Availability Statement

All datasets presented in this study are included in the article/supplementary material.

## Author Contributions

LG designed and performed the study and experiments, analyzed the data, and drafted the manuscript. YG and JL designed the study and experiments and analyzed the data. KW and ZZ helped to draft and revise the manuscript. SY was responsible for methodology, software, and analyzed the data. NJ and YZ contributed to project administration. SL took charge of conceptualization and funding acquisition. All authors contributed to the article and approved the submitted version.

## Conflict of Interest

The authors declare that the research was conducted in the absence of any commercial or financial relationships that could be construed as a potential conflict of interest.

## Footnotes

1 http://www.dichroweb.cryst.bbk.ac

2 https://swissmodel.expasy.org/

3 https://servicesn.mbi.ucla.edu/SAVES/

4 https://www.pymol.org
