# Corrigendum: Directed Evolution of *Pseudomonas fluorescens* Lipase Variants With Improved Thermostability Using Error-Prone PCR

**DOI:** 10.3389/fbioe.2020.602138

**Published:** 2020-10-23

**Authors:** Lijun Guan, Yang Gao, Jialei Li, Kunlun Wang, Zhihong Zhang, Song Yan, Nina Ji, Ye Zhou, Shuwen Lu

**Affiliations:** Institute of Food Processing, Heilongjiang Academy of Agricultural Sciences, Harbin, China

**Keywords:** lipase, structural analysis, site-directed mutagenesis, thermostability, methanol tolerance

In the published article, there was an error in the affiliation. Instead of “Institute of Food Processing, Heilongjiang Academy of Sciences, Harbin, China,” it should be “Institute of Food Processing, Heilongjiang Academy of Agricultural Sciences, Harbin, China.”

The authors apologize for this error and state that this does not change the scientific conclusions of the article in any way. The original article has been updated.

